# The outcomes of hybrid virtual consultation for a smoking cessation program in Klang Valley, Malaysia

**DOI:** 10.18332/tid/200071

**Published:** 2025-04-07

**Authors:** Nurkhaledatul Falah Bin Nuzma Adil, Jaya Kumar Murthy, Isa Bin Naina Mohamed, Teh Rohaila Binti Jamil, Rashidi Mohamed Bin Pakri Mohamed

**Affiliations:** 1 Department of Family Medicine, Faculty of Medicine, Universiti Kebangsaan Malaysia, Kuala Lumpur, Malaysia; 2 Department of Physiology, Faculty of Medicine, Universiti Kebangsaan Malaysia, Kuala Lumpur, Malaysia; 3 Department of Pharmacology, Faculty of Medicine, Universiti Kebangsaan Malaysia, Kuala Lumpur, Malaysia

**Keywords:** smoking cessation, quit smoking, virtual consultation, telemedicine, teleconsultation

## Abstract

**INTRODUCTION:**

Smoking has become a leading preventable cause of premature death and morbidity worldwide, with 8 million people dying each year because of tobacco. In Malaysia, a 24-week standard smoking cessation program is available to help smokers. Teleconsultation was introduced into this program during the COVID-19 pandemic by using internet-based video counseling to reduce the number of clinic visits. This study aimed to evaluate the outcomes of hybrid virtual consultation for smoking cessation programs among patients with nicotine dependence.

**METHODS:**

A retrospective cross-sectional study was conducted where all the active smokers registered in the smoking cessation program from 2018 to 2023 were recruited. They were grouped into face-to-face interventions and hybrid virtual consultations. All data were obtained from the smoking cessation program registry. The primary outcome was point abstinence (PA) at week 7 (1-month post-quit date), biochemically verified with carbon monoxide (CO) Smokerlyzer for both face-to-face and hybrid groups.

**RESULTS:**

A total of 156 participants were included in this study, including face-to-face (99 participants) and hybrid virtual consultation (57 participants). The mean age of face-to-face and hybrid group participants was 51 and 48 years, respectively. In general, hybrid virtual consultation was more feasible, as evidenced by a lower defaulter rate and a higher rate of participants graduating at the end of the program than face-to-face consultation. The effectiveness of smoking cessation was also higher in hybrid consultation, with a higher abstinence rate at weeks 4 and 7, with percentages of 42.1% and 56.1%, respectively. Additionally, the hybrid group maintained a high continuous abstinence rate (CAR) from week 7 to 24, with a percentage of 56.1%.

**CONCLUSIONS:**

Hybrid virtual consultation was more effective, as evidenced by higher smoking cessation at week 7 (1-month post-quit date) and CAR from week 7 to week 24 compared to the face-to-face group. Telemedicine or teleconsultation should be easily available for smoking cessation programs, and healthcare providers should consider incorporating hybrid models into them to fully utilize the program and improve outcomes.

## INTRODUCTION

According to the World Health Organization (WHO), smoking has become the leading preventable cause of premature death and morbidity worldwide^1^. It has been reported that globally, 8 million people die each year because of tobacco, with 1.2 million of them being non-smokers exposed to secondhand smoke^2^. The prevalence of smoking globally is decreasing in trend due to multiple tobacco control measures implemented in many countries. In 2020, the prevalence of smokers globally was 22.3%, with 36.7% of males and 7.8% of females smoking, slightly reduced from 2015, with a prevalence of 24.4%^1^. The prevalence is expected to decrease to 20.4% by 2025. In Malaysia, the prevalence of smokers is also declining, with 21.3% in 2019, down from 22.8% in 2015, including 40.5% of males and 1.2% of females^3^. The rate can be further reduced by a high-quality smoking cessation program aligned with the WHO Non-Communicable Diseases Global Target, which aims to decrease the smoking prevalence in our country by 15% before 2025 and <5% by 2040, in line with the target of National Strategic Plan 2021-2030, towards tobacco end game^4^. Several programs have been developed in Malaysia through the National Strategic Plan on Tobacco Control by the Ministry of Health (MOH) since 2015 to achieve this target. The smoking cessation program is one of the initiatives by MOH towards smoke-free countries, as targeted by WHO, and involves non-pharmacological and pharmacological interventions. A smoking cessation clinic is one of the initiatives conducted at health clinics, which medical officers typically conduct through physical consultation. The treatment varies, ranging from simple advice to extensive therapies with pharmacotherapy to ensure the effectiveness of smoking cessation. Since the COVID-19 pandemic, telemedicine has been widely used in healthcare services, including for smoking cessation^5^. Telemedicine involves the application of electronic information and communications technologies (ICT) to provide and support healthcare when distance separates the participants^6^. Globally, telemedicine has been adopted in smoking cessation programs, including providing multiple smoking cessation websites and virtual consultations. Studies have shown that the internet is an appealing platform to reach smokers due to its low cost and its ability to support limited healthcare availability and to avoid stigmatization^7^.

In Malaysia, a recent study showed that almost 90% of smokers attempted to quit smoking during COVID-19, with 60.1% intended to quit smoking. However, half of the respondents were unaware of the Quitline service provided by the government^8^. Thus, to achieve the target from the National Strategic Plan on Tobacco Control 2021-2030, it is important to create awareness of telemedicine availability and provide easy access to smoking cessation programs among smokers. Among the smokers, 37.1% agreed that smoking cessation websites were good, and 34.2% found them helpful for quitting smoking. However, frequent follow-up and motivation play an important role in ensuring sustainability^9^. Compliance with follow-up can be improved through teleconsultation, as the patients can attend the session virtually from everywhere and save time. Hybrid virtual consultation has been introduced into the smoking cessation program since the COVID-19 pandemic to sustain the accessibility of the service.

To date, the efficacy of virtual consultation for smoking cessation in Malaysia has not been previously reported. Determining whether virtual consultation can be implemented as a permanent service for the program is important. This study aimed to determine the efficacy and feasibility of hybrid virtual consultation for smoking cessation clinics. The outcome of this study can become a critical determinant of whether hybrid virtual consultation should be continued and maintained as a permanent service in the future, beyond the COVID-19 pandemic, for smoking cessation clinics in Malaysia.

## METHODS

### Study overview

This was a retrospective cross-sectional study to assess the outcomes of hybrid virtual teleconsultation for a smoking cessation program conducted in Primary Care Clinic, National University of Malaysia, Kuala Lumpur. Among participants in the program, we compared the success rates of smoking cessation between smokers attending face-to-face and hybrid virtual consultations. Ethics approval was obtained from the Research Ethics Committee, National University of Malaysia.

### Participants

Active smokers aged ≥18 years registered in the smoking cessation program in Primary Care Clinic, National University of Malaysia, Kuala Lumpur, between January 2018 and December 2023. All the data were obtained from the smoking cessation clinic registry, and all participants were previously interviewed on demographic background, smoking status, including number of cigarettes per day, and motivation level to quit. The participants were grouped into face-to-face only consultation and hybrid virtual consultation. Those with incomplete data due to missing follow-up during the program were excluded from the analysis.

### Smoking cessation program and procedures

**Figure 1:**
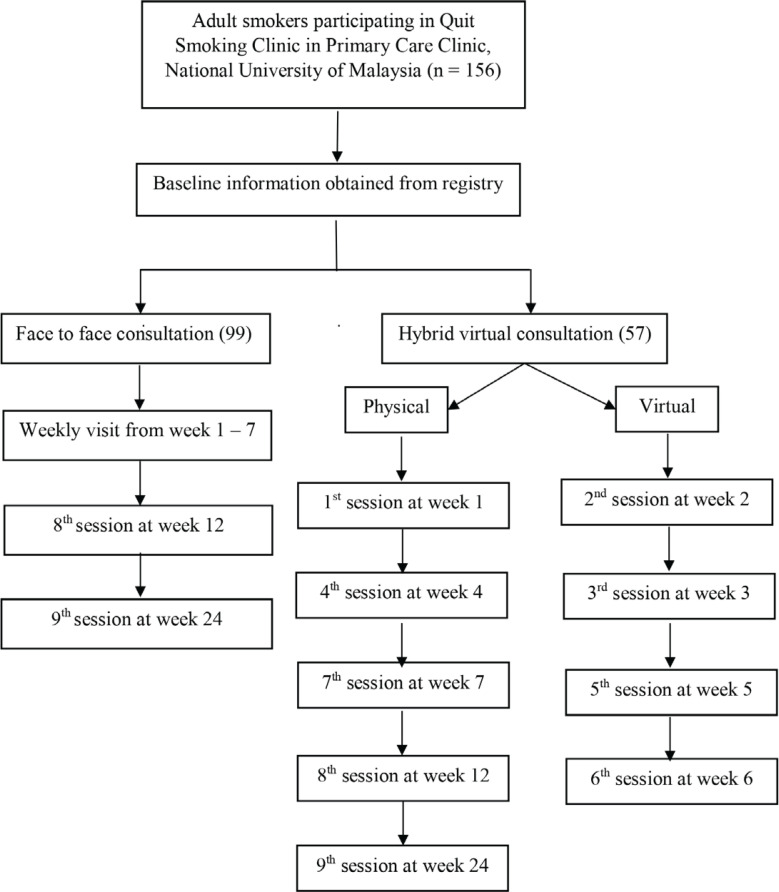
Flow chart of follow-up for smoking cessation program in Primary Care Clinic, National University of Malaysia

The smoking cessation program consists of a structured 24-week plan for both standard face-to-face consultation and hybrid virtual consultation. All participants visited the clinic on their initial visit (week 1) for registration and an explanation regarding the program. During this visit, participants were introduced to smoking cessation methods, their motivation to quit smoking was assessed using a motivation level questionnaire by the National Health Survey (NHS), their nicotine dependence was evaluated using the Fagerström test for nicotine dependence, and their expired breath carbon monoxide (CO) level was measured with a CO Smokerlyser^10^. Additionally, physicians conducted some assessments and decided on the prescription for nicotine replacement therapy (NRT) if needed. For week 2, the participants needed to set a quit date and prepare themselves for complete abstinence. In week 3, participants were expected to prepare for the quit day and discuss topics related to nicotine withdrawal and methods to overcome withdrawal symptoms. From weeks 4 to 6, participants shared their experiences during the smoking abstinence period, and later in week 7, they were provided with information on relapse prevention. The standard face-to-face consultation consists of nine clinic visits over 24 weeks, including doctor consultations and exhaled CO measurements using a CO Smokerlyser during each visit. The participants were seen weekly from week 1 to week 7, week 12, and week 24 (a total of 9 visits). At each visit, the participants reported abstinence verbally, and it was verified biochemically with a CO concentration (ppm) value measured by CO Smokerlyser. They also received consultation from a medical officer at each visit. For the hybrid virtual consultation group, in a total of nine sessions, five sessions were conducted via classical face-to-face consultation, and another four sessions were conducted virtually. The participants received consultation through video teleconsultation via Google Meet at week 2, week 3, week 5, and week 6 (4 sessions). A physical clinic visit was required at week 1, week 4, week 7, week 12, and week 24 (5 sessions) as the abstinence needed to be verified biochemically with CO smokerlyser. After week 24, all participants were discharged or graduated depending on their smoking status (Figure 1).

### Outcomes

We aimed to demonstrate the feasibility and efficacy of hybrid virtual consultation for smoking cessation programs. The primary outcome was point abstinence (PA) at week 7 (1 month post-quit date). The secondary outcomes were PA at week 4 (1week post-quit date) and the continuous abstinence rate (CAR) from week 7 to week 24. Smoking cessation success or point abstinence was defined as self-reported 4-week abstinence if the participant was assessed face-to-face 4 weeks after the designated quit date and declared that they had not smoked even a single puff on a cigarette in the past 30 days. Their expired CO assessed 4 weeks after the designated quit date was <10 ppm (Russell Standard, Clinical)^10^. CAR was defined as <5 cigarettes over the past 6 months as one or more consecutive weeks of smoking cessation successes since the program session finished at week 24. If participants declared that they had point abstinence at both weeks 7 and 24 during follow-up that was verified biochemically with the CO smokerlyser (<10 ppm), the participants were considered to have achieved continuous abstinence from weeks 7 to 24 (CAR7-24)^11^. Participants who self-reported ‘relapse’ or did not appear during follow-up (‘no reply’) were considered to have failed smoking cessation. Participants were considered discharged if they relapsed or were not ready to quit smoking throughout the programs.

### Sample size

The sample size for this study was calculated using G*Power 3.1.9.4 software based on the findings of a randomized controlled trial (RCT) that compared the efficacy of an internet-based remote smoking cessation program (telemedicine) with the standard face-to-face clinical visit program among patients with nicotine dependence^12^. The reference study reported similar continuous abstinence rates (CARs) from weeks 9 to 12 between the telemedicine group (81.0%) and the face-to-face group (78.9%), with an absolute difference of 2.1% (95% CI: -12.8–17.0). The study concluded that the telemedicine program was not inferior to the standard face-to-face program^12^. A one-tailed Z-test for proportions was performed to compare the continuous abstinence rates (CARs) between the control group (78.9% CAR) and the intervention group (81.0% CAR). The significance level (α) was 0.05, and the power (1-β) was 0.80. The calculation yielded a required sample size of 58 participants per group (total=116). To account for a potential 20% dropout rate, the sample size was adjusted to 73 participants per group (total=146). This ensures adequate power to confirm the non-inferiority of hybrid virtual consultation compared to face-to-face consultation.

### Data collection

We collected baseline information and follow-up details on each participant through the registry of the Quit Smoking Clinic of the Primary Care Clinic, National University of Malaysia. The baseline information includes their age, gender, race, education level, number of cigarettes per day, Fagerström test for nicotine dependence (FTND) score, and motivation level. The follow-up details include the number of cigarettes smoked per day, smoking status, and CO level. For FTND, the participants were categorized into different levels of nicotine dependence, ranging from low to high dependence, according to their total score. Higher scores indicate greater nicotine dependence and a more severe addiction to smoking (0–3 low, 4–6 moderate, and 7–10 high). CO level was measured using a smokerlyzer device to identify the level of carbon monoxide in the body and to determine the zone they fall in. The level was divided into three color zones: green (1–6 ppm), amber (7–10 ppm), and red (>10 ppm) that indicate non-smoker, light-smoker or passive smoker, and heavy smoker, respectively. The motivation level of participants was measured using the Motivation to Quit Smoking by the National Health Survey, which consists of two questions^13^. The first question was: ‘How important is it to you to give up smoking altogether at this attempt?’ with responses ‘4 - desperately important’, ‘3 - very important’, ‘2 - quite important’, and ‘1 - not all that important’. The second question was: ‘How determined are you to give up smoking at this attempt?’ with responses ‘4 - extremely determined’, ‘3 - very determined’, ‘2 - quite determined’, and ‘1 - not all that determined’. Responses for both questions were totaled from 2 to 8, with a higher score indicating a higher overall motivation level.

### Statistical analysis

Descriptive and inferential data were analyzed using IBM SPSS Statistics version 27 (IBM Corp., Armonk, NY, USA). Continuous variables were presented as mean ± standard deviation (SD), and categorical variables were presented as frequencies and percentages. All statistical tests were two-tailed, and a p<0.05 was considered statistically significant. The normality of the data was assessed using the Kolmogorov-Smirnov and Shapiro-Wilk tests, which indicated that all variables were non-normally distributed (p<0.05). As a result, non-parametric tests were used for preliminary analyses. Potential confounders were identified based on two criteria: 1) significant differences between groups at baseline (face-to-face vs hybrid); and 2) significant association with the outcome (number of cigarettes smoked per day and CO level). Baseline differences between the face-to-face and hybrid groups were assessed using the Mann-Whitney U test for continuous variables (e.g. age, nicotine dependence) and the Fisher’s exact test for categorical variables (e.g. treatment, education level).

The association between potential confounders and the outcomes (number of cigarettes smoked per day and CO level) was assessed using Spearman correlation due to the non-normal distribution of the data. Based on these correlations, nicotine dependence (FTND), treatment (NRT), motivation to quit smoking, and education level were included as covariates in the final model. Nicotine dependence (FTND) and treatment (NRT) were included because they met both criteria for confounders (significant baseline differences and association with the outcomes). Motivation to quit smoking and education level were included because they showed significant associations with the number of cigarettes smoked per day, even though they did not differ significantly between groups at baseline. These variables were considered important predictors of smoking behavior and were included to improve the robustness of the analysis. Age, although differing significantly between groups at baseline (p=0.046), was excluded as a confounder because it was not significantly associated with either outcome (all p>0.05). Gender and race were also excluded because they differed significantly between groups and were not associated with the outcomes.

A repeated measures ANCOVA was conducted to examine the effects of Time (within-subjects factor: Week 1, Week 4, and Week 7) and Group (between-subjects factor: face-to-face vs hybrid) on the number of cigarettes smoked per day and CO level while controlling for nicotine dependence (FTND), treatment (NRT), motivation to quit smoking, and education level as covariates. *Post hoc* tests with Bonferroni correction were conducted to explore pairwise differences. Finally, the chi-squared test was used to evaluate the association between abstinence rate and method of consultation.

## RESULTS

We retrospectively analyzed 156 participants from a smoking cessation clinic between January 2018 and December 2023. All participants were included for further analysis (Figure 1), with 99 participants in the face-to-face consultation group and 57 participants in the hybrid virtual consultation group. The demographic and baseline characteristics of the participants are summarized in Table 1. The mean age was 51 ± 13.3 years for the face-to-face group and 48 ± 12.0 years for the hybrid group, with a statistically significant difference between the two groups (p=0.046). Both groups predominantly consisted of male participants (face-to-face: 94.9%; hybrid: 94.7%) and were of Malay ethnicity (face-to-face: 65.6%; hybrid: 73.7%). For the education level, most participants in the hybrid group had tertiary education (59.7%). In contrast, the face-to-face group had a more even distribution, with 48.5% having secondary education and 45.5% having tertiary education (p=0.225). The number of cigarettes smoked per day (face-to-face: 12.4 ± 0.8; hybrid: 12.3 ± 1.1; p=0.981), FTND scores (face-to-face: 4.9 ± 2.4; hybrid: 4.3 ± 2.1; p=0.080), and motivation to quit (face-to-face: 6.3 ± 1.8; hybrid: 6.9 ± 0.9; p=0.127) were similar in both groups. Both groups primarily used nicotine-based pharmacotherapy (NRT), with slightly higher usage in the hybrid group (96.5% vs 79.8%; p=0.004). No participants were prescribed varenicline in either group.

**Table 1:** Sociodemographic and baseline characteristics of participants of smoking cessation from 2018 to 2023 (N=156)

*Characteristics*	*Face-to-face* *(N=99)* *n (%)*	*Hybrid* *(N=57)* *n (%)*	*p*
**Age** (years), mean ± SD	51 ± 13.3	48 ± 12.0	0.046
**Gender**			
Male	94 (94.9)	54 (94.7)	1.000
Female	5 (5.1)	3 (5.3)	
**Race**			
Malay	65 (65.6)	42 (73.7)	0.388
Chinese	25 (25.3)	13 (22.8)	
Indian	9 (9.1)	2 (3.5)	
**Education level**			
Primary	6 (6.0)	2 (3.5)	0.225
Secondary	48 (48.5)	21 (36.8)	
Tertiary	45 (45.5)	34 (59.7)	
**Cigarettes/day,** mean ± SD	12.4 ± 0.8	12.3 ± 1.1	0.981
**FTND score**^a^, mean ± SD	4.9 ± 2.4	4.3 ± 2.1	0.080
**Motivation to quit,** mean ± SD	6.3 ± 1.8	6.9 ± 0.9	0.127
**Nicotine-based pharmacotherapy** (NRT)			
Yes	79 (79.8)	55 (96.5)	0.004
No	20 (20.2)	2 (3.5)	

a FTND: Fagerström test for nicotine dependence. All continuous variables were compared using the Mann-Whitney U test. All categorical variables were compared using Fisher’s exact test.

### Retention rate and overall success rate for both face-to-face and hybrid virtual consultation

Table 2 presents the attendance rates and success rates of participants in the smoking cessation program, comparing face-to-face and hybrid virtual consultation groups. The results showed that a significantly higher percentage of participants in the hybrid group attended >80% of sessions (59.6%) compared to the face-to-face group (30.3%) (χ^2^=13.845, p<0.001). Conversely, a higher proportion of participants in the face-to-face group attended ≤80% of sessions (69.7%) compared to the hybrid group (40.6%), indicating better adherence in the hybrid model. In terms of program success, the hybrid group also demonstrated a higher graduation rate (66.7%) compared to the face-to-face group (46.0%) (χ^2^=4.510, p=0.034). Additionally, the discharge rate was higher in the face-to-face group (54.0%) than in the hybrid group (33.3%), further highlighting the feasibility and effectiveness of the hybrid virtual consultation approach.

**Table 2:** The feasibility of hybrid virtual consultation for smoking cessation clinic from 2018 to 2023 (N=156)

*Variables*	*Face-to-face* *(N=99)* *n (%)*	*Hybrid* *(N=57)* *n (%)*	*χ^2^*	*p*
**Number of sessions attended**				
>80	30 (30.3)	34 (59.6)	13.845	<0.001*
≤80	69 (69.7)	23 (40.6)		
**Retention rate**				
Graduated	29 (46.0)	30 (66.7)	4.510	0.034*
Discharged	34 (54.0)	15 (33.3)		

* The chi-squared test was used to compare proportions between the face-to-face and hybrid groups. A p<0.05 indicates statistical significance. Retention rates are based on participants who graduated or were discharged.

### Number of cigarettes smoked per day

For the number of cigarettes smoked per day, nicotine dependence (FTND) showed a strong positive correlation at all time points (ρ=0.589, p<0.001). In contrast, motivation to quit showed a strong negative correlation (ρ= -0.495, p<0.001). Education level was weakly negatively correlated with smoking behavior in Week 1 (ρ= -0.196, p=0.014) and Week 4 (ρ= -0.180, p=0.024). Treatment (NRT) showed a weak negative correlation at Week 4 (ρ= -0.202, p=0.011). For CO level, nicotine dependence (FTND) showed a weak positive correlation at Week 1 (ρ=0.188, p=0.019) and weak negative correlations at Week 7 (ρ= -0.210, p=0.008). Treatment (NRT) showed a weak positive correlation with CO level at Week 1 (ρ=0.230, p=0.004).

**Figure 2:**
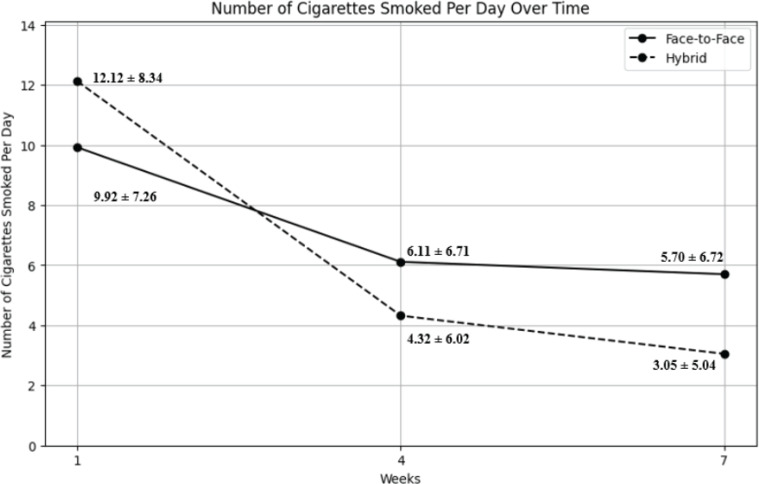
Trends in number of cigarettes smoked per day in face-to-face and hybrid group at week 1, 4 and 7 of smoking cessation clinic

The number of cigarettes smoked per day showed a decreasing trend over time in both the face-to-face and hybrid groups, as shown in Figure 2. In the face-to-face group, the mean number of cigarettes smoked per day decreased from 9.92 ± 7.26 in Week 1 to 6.11 ± 6.71 in Week 4 and 5.70 ± 6.72 in Week 7. Similarly, in the hybrid group, the mean number of cigarettes smoked per day decreased from 12.12 ± 8.34 in Week 1 to 4.32 ± 6.02 in Week 4 and 3.05 ± 5.04 in Week 7. Table 3 presents the repeated measures of the ANCOVA results, which were adjusted using the Greenhouse-Geisser correction. The within-group contrasts revealed significant reductions in cigarette consumption from Week 1 to Week 4 and Week 1 to Week 7 in both groups (all p<0.001). However, the reduction from Week 4 to Week 7 was only significant in the hybrid group (p<0.001), while the face-to-face group showed no significant change during this period (p=0.647). Between-group comparisons indicated a significant difference in cigarette consumption at Week 1 (p<0.001), with the hybrid group smoking more cigarettes than the face-to-face group. However, there were no significant differences between the groups in Week 4 (p=0.926) or Week 7 (p=0.184).

**Table 3:** The number of cigarettes smoked per day in face-to-face and hybrid group at week 1, 4 and 7 of smoking cessation clinic (N=156)

*Time point*	*Group*	*Cigarettes/day* *Mean ± SD*	*Within-group contrasts (p)*	*Between-group contrasts (p)*	*Group×Time interaction (F, p)*	*Effect size (partial η^2^)*
Week 1	Face-to-Face	9.92 ± 7.26	Week 1 vs Week 4 <0.001	Week 1 <0.001	17.65 <0.001	0.105
	Hybrid	12.12 ± 8.34	Week 1 vs Week 4 <0.001			
Week 4	Face-to-Face	6.11 ± 6.71	Week 4 vs Week 7 <0.001	Week 4 0.926		
	Hybrid	4.32 ± 6.02	Week 4 vs Week 7 0.647			
Week 7	Face-to-Face	5.70 ± 6.72	Week 1 vs Week 7 <0.001	Week 7 0.184		
	Hybrid	3.05 ± 5.04	Week 1 vs Week 7 <0.001			

The repeated measures ANCOVA model included the following covariates: nicotine dependence (FTND), treatment (NRT), motivation to quit smoking, and education level. The within-subjects factor was Time (Week 1, Week 4, Week 7), and the between-subjects factor was Group (face-to-face vs hybrid). Both within-group and between-group comparisons are Bonferroni-adjusted. Box’s test of equality of covariance matrices indicated a violation of the homogeneity of covariance matrices assumption (Box’s M=15.267, p=0.021). Multivariate tests using Pillai’s trace confirmed a significant Time×Group interaction (p<0.001), but the main effect of Time [F(2; 149)=1.575, p=0.210] and between-subjects effect of Group [F(1, 150)=1.31, p=0.254] were not significant. Mauchly’s test of sphericity [W=0.326, χ^2^(2)=167.107, p<0.001] indicates that the assumption of sphericity was violated. Therefore, the Greenhouse-Geisser correction (ε=0.597) was applied.

The analysis revealed a significant Time×Group interaction [F(1.195; 179.188)= 17.65, p<0.001, partial η^2^=0.105], indicating that the rate of reduction in cigarette consumption differed between the face-to-face and hybrid groups over time. Specifically, the hybrid group significantly reduced cigarette consumption compared to the face-to-face group. However, the main effect of time was insignificant [F(1.195; 179.188)=2.43, p=0.115, partial η^2^=0.016], suggesting that the overall reduction in cigarette consumption over time was not significant after controlling for covariates. The between-subjects effect of group was also not significant [F(1; 150)=1.31, p=0.254, partial η^2^=0.009], indicating no overall difference in cigarette consumption across all time points between the two groups. Among the covariates, nicotine dependence (p<0.001), motivation to quit (p=0.001), and education level (p=0.043) had significant effects on cigarette consumption, while nicotine-based pharmacotherapy (NRT) did not (p=0.429).

### Carbon monoxide levels (ppm)

**Figure 3:**
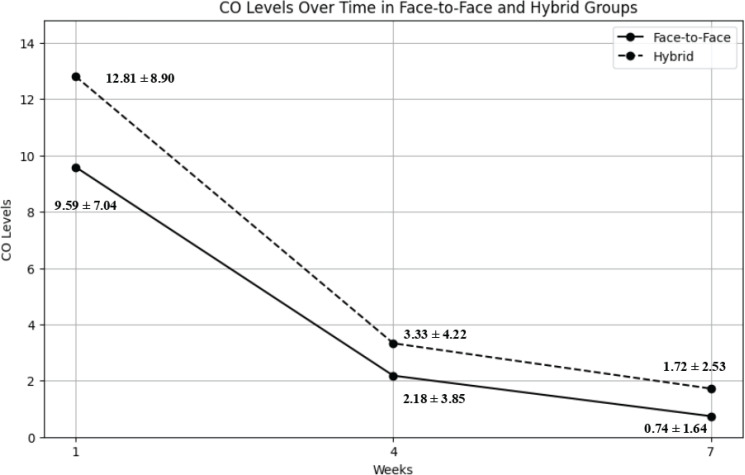
Trends in CO level (ppm) in face-to-face and hybrid group at week 1, 4 and 7 of smoking cessation clinic

Carbon monoxide (CO) levels decreased over time in face-to-face and hybrid groups, as shown in Figure 3. In the face-to-face group, the mean CO level decreased from 9.59 ± 7.04 ppm at Week 1 to 2.18 ± 3.85 ppm at Week 4 and 0.74 ± 1.64 ppm at Week 7. Similarly, in the hybrid group, the mean CO level decreased from 12.81 ± 8.90 ppm at Week 1 to 3.33 ± 4.22 ppm at Week 4 and 1.72 ± 2.53 ppm at Week 7. Table 4 presents the repeated measures of the ANCOVA results, which were adjusted using the Greenhouse-Geisser correction. The within-group contrasts revealed significant reductions in CO levels from Week 1 to Week 4 and Week 1 to Week 7 in both groups (all p<0.001). However, the reduction from Week 4 to Week 7 was significant in both the face-to-face group (p<0.001) and the hybrid group (p=0.017). Between-group comparisons indicated a significant difference in CO levels at Week 1 (p=0.027), with the hybrid group having higher CO levels than the face-to-face group. However, there were no significant differences between the groups in Week 4 (p=0.165) or Week 7 (p=0.003).

**Table 4:** Carbon monoxide (CO) levels in face-to-face and hybrid groups at week 1, 4, and 7 of smoking cessation clinic (N=156)

*Time point*	*Group*	*CO (ppm)* *Mean ± SD*	*Within-group contrasts (p)*	*Between-group contrasts (p)*	*Group×Time interaction (F, p)*	*Effect size (partial η^2^)*
Week 1	Face-to-Face	9.59 ± 7.04	Week 1 vs Week 4 <0.001	Week 1 0.027	1.927 0.161	0.013
	Hybrid	12.81 ± 8.90	Week 1 vs Week 4 <0.001			
Week 4	Face-to-Face	2.18 ± 3.85	Week 1 vs Week 7 <0.001	Week 4 0.165		
	Hybrid	3.33 ± 4.22	Week 1 vs Week 7 <0.001			
Week 7	Face-to-Face	0.74 ± 1.64	Week 4 vs Week 7 <0.001	Week 7 0.003		
	Hybrid	1.72 ± 2.53	Week 4 vs Week 7 0.017			

The repeated measures ANCOVA model included the following covariates: nicotine dependence (FTND), treatment (NRT), motivation to quit smoking, and education level. The within-subjects factor was Time (Week 1, Week 4, Week 7), and the between-subjects factor was Group (face-to-face vs hybrid). Both within-group and between-group comparisons are Bonferroni-adjusted. Box’s test of equality of covariance matrices indicated a violation of the homogeneity of covariance matrices assumption (Box’s M=18.478, p=0.006). Multivariate tests using Pillai’s trace confirmed a non-significant Time×Group interaction (p=0.311) and main effect of Time (p=0.572). Mauchly’s test of sphericity [W=0.572, χ^2^(2)=83.123, p<0.001] indicates that the assumption of sphericity was violated. Therefore, the Greenhouse-Geisser correction (ε=0.700) was applied.

The analysis revealed a non-significant Time×Group interaction (F=1.927, p=0.161, partial η^2^=0.013), indicating that the rate of reduction in CO levels did not differ significantly between the face-to-face and hybrid groups over time. The main effect of time was also not significant (F=0.209, p=0.730, partial η^2^=0.001), suggesting that the overall reduction in CO levels over time was not significant after controlling for covariates. However, the between-subjects effect of group was significant (F=7.759, p=0.006, partial η^2^=0.049), indicating that the hybrid group had higher CO levels overall compared to the face-to-face group across all time points. Among the covariates, nicotine dependence (FTND) had a significant effect on CO levels (F=9.103, p<0.001, partial η^2^=0.057), while treatment (NRT) (F=4.006, p=0.033, partial η^2^=0.026), motivation to quit smoking (F=0.627, p=0.480, partial η^2^=0.004), and education level (F=0.381, p=0.609, partial η^2^=0.003) did not show significant effects.

### Cigarette smoking status

Both groups demonstrated a reduction in the percentage of participants smoking over time (Table 5). In the face-to-face group, the percentage of participants not smoking increased slightly from 32.3% in Week 4 to 35.4% in Week 7. In contrast, the hybrid group showed a more substantial increase in the percentage of participants not smoking, rising from 42.1% in Week 4 to 56.1% in Week 7. In Week 1, there was no significant difference in smoking status between the face-to-face and hybrid groups (84.8% vs 91.2%, χ^2^=1.317, p=0.251). However, by Week 7, a significant difference emerged, with a higher percentage of participants in the hybrid group not smoking than the face-to-face group (43.9% vs 64.6%, χ^2^=6.379, p=0.012).

**Table 5:** Smoking status of participants and in face-to-face and hybrid group at week 1, 4, and 7 of smoking cessation clinic (N=156)

*Variables*	*Smoking status, n (% Yes)*		
	*Week 1*	*Week 4*	*Week 7*
Face-to-Face	84 (84.8)	67 (67.7)	64 (64.6)
Hybrid	52 (91.2)	33 (57.9)	25 (43.9)
Statistics	χ^2^=1.317 p=0.251	χ^2^=1.504 p=0.220	χ^2^=6.379 p=0.012

With regard to continuous abstinence rate (CAR) from Week 7 to Week 24 for both groups, marking the period during which participants were considered to have graduated from the smoking cessation clinic, the percentage of participants who maintained continuous abstinence was significantly higher in the hybrid group (56.1%) compared to the face-to-face group (29.3%) over the same period (χ^2^=10.86, p<0.001).

## DISCUSSION

This study demonstrated the overall outcomes of face-to-face and hybrid virtual consultation in smoking cessation clinics. The outcomes can be further classified into the feasibility and efficacy of the consultations. In general, we found that: 1) hybrid virtual consultation was more feasible, with a higher percentage of participants attending >80% of the session and a lower percentage of participants defaulting or being discharged from the program; 2) the retention rate was higher in hybrid virtual consultation, as evidenced by higher percentage of participants maintaining non-smoking status at week 24; and 3) the hybrid virtual consultation was more effective, with a higher percentage of participants graduating compared to face-to-face group.

As for the demographic background, most participants were males of Malay ethnicity, similar to the previous study with a higher prevalence of male smokers (51.6%) of Malay ethnicity (49.6%)^14^. The mean age was 51 years for the face-to-face group and 48 years for hybrid virtual consultation group. This differs from previous studies where most smokers were aged 25–44 years^14^. Most of the participants of this study were male, with only 5.1% female smokers, corresponding to the 1.2% prevalence of female smokers in Malaysia^3^. The success rate for smoking cessation was influenced by the level of nicotine dependence and the motivation level to quit. In this study, the overall level of nicotine dependence was low to moderate for both groups, consistent with a previous study done in Malaysia, where most smokers had low to moderate levels of nicotine dependence^15^. Additionally, having a tertiary education level is associated with a higher success rate of smoking cessation^16^. Most participants in this study for the hybrid group had a tertiary level of education, and the success rate was high in this group, correlating with findings reported from previous studies^16^.

There were several important findings from this study. First, this study suggested that hybrid virtual consultations for smoking cessation may result in better session attendance, with 59.6% attending >80% of sessions from the hybrid group and 30.3% from the face-to-face group, and higher graduation rates in the hybrid group (52.6%) compared to traditional face-to-face consultations (29.3%). Previous studies have shown low physical attendance rates for smoking cessation clinics, with only 10.5% of participants attending seven or more sessions^17^. Conversely, based on the cohort study in Japan, the dropout rate till week 24 was low, contributing to an overall high success rate in smoking cessation^12^. In this study, the retention rate was notably higher in the hybrid group, with 56.1% of CAR from week 7 to 24, indicating the potential benefits of incorporating virtual elements into smoking cessation programs. Compared to a randomized controlled trial done in Japan, the retention rate was relatively high in both face-to-face and telemedicine groups, with a 74.1% continuous abstinence rate from week 9 to 24^12^.

Second, the point abstinence rate at week 7 (1-month post-quit date) was relatively high in both groups (32.3% in the face-to-face group and 42.1% in the hybrid group). This study showed that while both interventions effectively reduced smoking rates over time, the hybrid group appeared more effective at week 7 and the end of the program (week 24). The significant difference in smoking status at week 7 suggested that the hybrid approach may offer additional benefits over the face-to-face method in the context of smoking cessation. A weekly follow-up till one month post-quitting leads to high cessation rates, as proven by this study, where the point abstinence rate for the hybrid group is higher at week 7 than at week 24, with fewer participants relapsing at the end of the program. However, there was no significant difference in the face-to-face group smoking cessation rate at week 4 and week 7, most likely because of the high rate of default weekly follow-up. These findings correspond to results shown by the previous studies where weekly follow-up in the first four weeks of quitting leads to a high cessation rate^18^. The first four weeks after quitting were crucial and challenging because of the high risk of withdrawal symptoms causing relapse^19^.

However, withdrawal symptoms can be prevented or treated with nicotine replacement therapy (NRT). In this study, the use of nicotine replacement therapy was high in both groups. However, the use in the hybrid group was higher, with 96.5% of participants starting on NRT. None of the participants in both groups was prescribed non-NRT (varenicline). A previous study mentioned that pharmacotherapy with NRT was prescribed to most of the participants, and <20% of participants were prescribed with non–NRT (varenicline)^18^. A minority of the participants refused NRT in the early part of the program, as seen in literature, due to several reasons such as concern about safety, perception of being addicted to it, and also fear of side effects^20^. Thus, the use of NRT will also influence the cessation rates. A systemic review found that NRT was relatively safe to be used in most patients for the short-term, but skin reaction, gastrointestinal upset, and palpitation were common^20^. In terms of the effectiveness of pharmacotherapy, a combination of nicotine gum and a patch was preferred to reduce the severity of withdrawal symptoms. Regardless of the treatment modalities, the rate of smoking cessation increased by 50–60% with NRT, as reported in Cochrane Library^21^.

Third, both face-to-face and hybrid smoking cessation methods were effective in reducing cigarette consumption over time. However, the hybrid group showed a more significant reduction by week 7, highlighting its potential as a more effective method for long-term smoking reduction. In week 7, the hybrid group smoked significantly fewer cigarettes than the face-to-face group, suggesting that the hybrid intervention was more effective. This could be related to the feasibility of hybrid consultation with a higher rate of compliance to follow-up because continuous counseling and monitoring were crucial to maintain the motivation to smoking cessation. Traditionally, there was a decreasing trend in the number of cigarettes smoked over time with proper counseling via face-to-face sessions either individually or in group therapy^11^. Besides that, both interventions effectively reduced CO levels over the 7 weeks. The hybrid group showed a more substantial reduction in CO levels by the end of the study, suggesting it might be more effective in reducing exposure to carbon monoxide from smoking. This finding indicates that while both face-to-face and hybrid smoking cessation programs are effective, the hybrid program might offer superior results in reducing CO levels among participants. This aligns with the earlier findings on the number of cigarettes smoked, further suggesting the potential greater efficacy of hybrid programs in smoking cessation efforts.

### Strengths and limitations

The strength of this study is that it was the first to evaluate the feasibility and efficacy of hybrid virtual consultation in smoking cessation clinics. However, there are a few limitations, including a small sample size with a limited number of participants, power considerations were not explicitly performed in the design of this study due to limited prior data on the expected effect size. Future studies should aim to include power calculations to optimize sample size and ensure adequate power to detect significant effects. Also, there was a limited number of female participants, which could impact the generalizability of the findings in other countries. This study also focuses only on the short-term outcomes of smoking cessation with a short period of follow-up. A more extended follow-up period is necessary to evaluate the outcomes of the intervention. It is also necessary to explore other factors that can influence the effectiveness of the intervention.

## CONCLUSIONS

This study evaluates the feasibility and efficacy of hybrid virtual consultation in a smoking cessation clinic that has potential benefits for future smoking cessation programs in Malaysia. Overall, the results demonstrated that both methods and approaches are effective for smoking cessation. However, further evaluation showed that hybrid virtual consultation appears to have greater flexibility and accessibility, leading to better adherence and significant smoking cessation. Within our context, telemedicine or teleconsultation should be readily available for smoking cessation programs, and healthcare providers should consider incorporating hybrid models into smoking cessation programs to fully utilize the program and better outcomes.

## Data Availability

The data supporting this research are available from the authors on reasonable request.
